# The cytohesin paralog Sec7 of *Dictyostelium discoideum is* required for phagocytosis and cell motility

**DOI:** 10.1186/1478-811X-11-54

**Published:** 2013-08-01

**Authors:** Rolf Müller, Claudia Herr, Salil K Sukumaran, Napoleon Nosa Omosigho, Markus Plomann, Tanja Y Riyahi, Maria Stumpf, Karthic Swaminathan, Marios Tsangarides, Kyriacos Yiannakou, Rosemarie Blau-Wasser, Christoph Gallinger, Michael Schleicher, Waldemar Kolanus, Angelika A Noegel

**Affiliations:** 1 Institute of Biochemistry I, Medical Faculty, Center for Molecular Medicine Cologne (CMMC) and Cologne Excellence Cluster on Cellular Stress Responses in Aging-Associated Diseases (CECAD), University of Cologne, 50931 Köln, Germany; 2 Institute of Biochemistry II, Medical Faculty, University of Cologne, 50931 Köln, Germany; 3 Institute of Anatomy and Cell Biology, Ludwig-Maximilians-University, 80336 München, Germany; 4 Laboratory of Molecular Immunology, LIMES Institute of the University of Bonn, Bonn, Germany

**Keywords:** ARFGEF, Cell adhesion, Cell migration, Phagocytosis, Phosphoinositide binding

## Abstract

**Background:**

*Dictyostelium* harbors several paralogous Sec7 genes that encode members of three subfamilies of the Sec7 superfamily of guanine nucleotide exchange factors. One of them is the cytohesin family represented by three members in *D. discoideum*, SecG, Sec7 and a further protein distinguished by several transmembrane domains. Cytohesins are characterized by a Sec7-PH tandem domain and have roles in cell adhesion and migration.

**Results:**

We study here Sec7. In vitro its PH domain bound preferentially to phosphatidylinositol 3,4-bisphosphate (PI(3,4)P_2_), phosphatidylinositol 4,5-bisphosphate (PI(4,5)P_2_) and phosphatidylinositol 3,4,5-trisphosphate (PI(3,4,5)P_3_). When following the distribution of GFP-Sec7 in vivo we observed the protein in the cytosol and at the plasma membrane. Strikingly, when cells formed pseudopods, macropinosomes or phagosomes, GFP-Sec7 was conspicuously absent from areas of the plasma membrane which were involved in these processes. Mutant cells lacking Sec7 exhibited an impaired phagocytosis and showed significantly reduced speed and less persistence during migration. Cellular properties associated with mammalian cytohesins like cell-cell and cell-substratum adhesion were not altered. Proteins with roles in membrane trafficking and signal transduction have been identified as putative interaction partners consistent with the data obtained from mutant analysis.

**Conclusions:**

Sec7 is a cytosolic component and is associated with the plasma membrane in a pattern distinctly different from the accumulation of PI(3,4,5)P_3_. Mutant analysis reveals that loss of the protein affects cellular processes that involve membrane flow and the actin cytoskeleton.

## Background

ADP ribosylation factor (ARF) GTPases have roles in vesicular transport, in the regulation of actin cytoskeleton dynamics, cell adhesion, cell migration and in signal transduction processes. They depend on specific nucleotide exchange factors, the ARFGEFs, which through their conserved Sec7 domain (Sec7d) catalyze the GDP to GTP exchange. Six subfamilies of ARFGEFs exist in eukaryotes, the large ARFGEFs of the GBF and BIG family, and the small ARFGEFs of the cytohesin, EFA6, BRAG and FBX family. For all ARFGEFs localization to membranes is important for their functions in ARF activation. BIG1 and BIG2 localize to the TGN and endosomes, GBF localizes to the Golgi, and cytohesins are found at the cell periphery where they function in plasma membrane endosomal membrane trafficking and in signal transduction pathways [1].

Cytohesins are composed of a Sec7d domain and a pleckstrin homology (PH) domain of the cytohesin type followed by a polybasic stretch which together with the PH domain is necessary for plasma membrane association and biological function [2]. The PH domain of the cytohesins specifically binds to phosphoinositides, whose levels are influenced by PI3-kinases in response to upstream signals. Upon recruitment to the plasma membrane cytohesins activate ARFs, which influence the cortical actin cytoskeleton, endocytic processes and phagocytosis. Cytohesins also regulate signaling pathways by functioning in scaffolding complexes. Such a complex was reported for cytohesin-2 which through its N-terminal sequences could assemble a protein complex that contained the RacGEF DOCK180 and promoted Rac activation and cell migration [3]. Furthermore, in dendritic cells cytohesin-1 regulated migration in vivo by functioning upstream of RhoA activation [4].

The lower eukaryote *D. discoideum* harbors six ARFGEFs belonging to three families, the GBF and BIG family with one and two representatives each, and three members of the cytohesin family harboring the characteristic Sec7-PH tandem [5]. The finding of this class of proteins in a lower eukaryote was surprising as cytohesins had been reported only for metazoans [6]. Instead of the typical coiled coil domain at the N-terminus, the *Dictyostelium* cytohesins have variable domains. SecG (DDB_G0287459) has several ankyrin repeats and DDB0233591 (DDB_G0279241) harbors four predicted transmembrane domains whereas the N-terminus of DDB0233617 (DDB_G0272486) which we designate Sec7 has no putative conserved domains [5]. Instead, homopolymer tracts of asparagine and threonine are present. Such homopolymer tracts are quite frequent in *D. discoideum* proteins [7,8].

In previous work we had analyzed the function of SecG and found that mutant cells lacking SecG had reduced cell-substratum adhesion whereas cell cell adhesion was not affected. In cell migration analysis speed was significantly reduced, persistence and directionality of migration were unaltered. Here we analyze the Sec7 protein and characterize Sec7 deficient cells. We find that Sec7 is a cytosolic component and is associated with the plasma membrane in a pattern distinctly different from the accumulation of PI(3,4,5)P_3._ We compare these data with the one reported for known PH sensors and ArfA, the single ARF GTPase of *D. discoideum*.

## Results

### Sec7 associates with the plasma membrane

**Figure 1:**
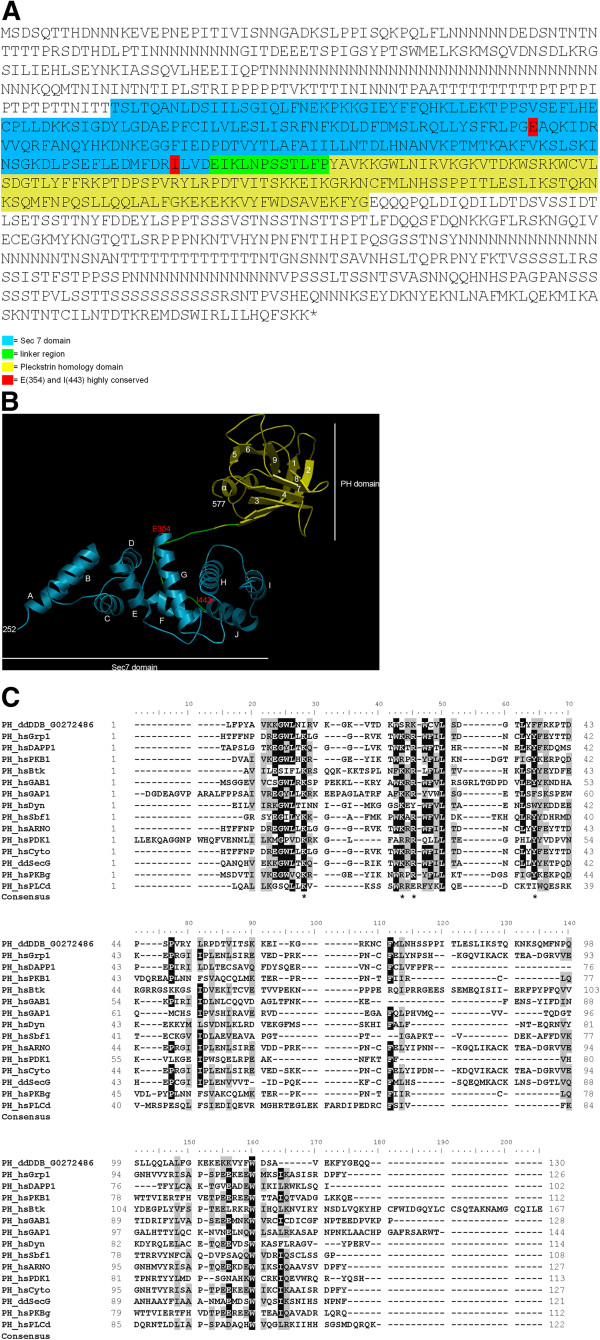
**Domain structure of the Sec7 protein. (A)** The amino acid sequence of Sec7 of *D. discoideum* is shown (DDB0233617). The Sec7 domain is highlighted in blue, the linker region in green and the Pleckstrin homology (PH) domain in yellow. The highly conserved amino acids E(354) and I(443) are marked with red. **(B)** The DDB0233617 Sec7-PH-domain modeled to 2r0dA (Crystal Structure of Autoinhibited Form of Grp1 Arf GTPase Exchange Factor). The Sec7 domain is depicted in blue, the linker region in green and the PH domain in yellow. The highly conserved amino acids E(354) and I(443) are shown in red. Modeling was with SwissModel, visualization with OpenAstexViewer. **(C)** CLUSTALX alignments of PH domains from *Homo sapiens* and *D. discoideum* according to Lietzke et al. and Ferguson et al. [11,12]. The asterisks in the Consensus line indicate the signature motif as suggested by Lietzke et al. and Ferguson et al. [11,12]. ddDDB_G0272486 corresponds to Sec7.

The *D. discoideum* cytohesin Sec7 is a 931 amino acids protein of 103.797 kDa and has a pI of 7.97. The rather unstructured N terminal region encompasses ~250 amino acids, the Sec7 domain extends from amino acids 252 to 441 and is separated from the basic PH domain (residues 454–577) through a short linker (Figure 1A). The structure of Sec7-PH has been obtained by homology modeling using 2r0dA (Crystal Structure of Autoinhibited Form of Grp1 Arf GTPase Exchange Factor (Cytohesin-3)) as template (Figure 1B). The Sec7 domain of the *D. discoideum* protein contains 10 alpha-helices, A to J. The helix J (position 428-438) is crucial for ARF binding and exchange. The Sec7 domain also includes the key residue glutamate (E) (position 354), which is essential for GDP to GTP exchange, and an isoleucine (I) at position 443 that is part of a hydrophobic cluster involved in ARF interaction (Figure 1B, indicated in red). Thus it corresponds to a typical Sec7 domain [9,10].

The PH domains of cytohesins bind polyphosphoinositides and have different affinities and specificities for PI(3,4)P_2_, PI(4,5)P_2_ and PI(3,4,5)P_3_ depending on their structure [2,13,14]. The signaling lipids PI(3,4)P_2_ and PI(3,4,5)P_3_ are rare components of the plasma membrane and are produced in response to a stimulus which then recruits the PH domain proteins to the membrane. PH domains have a conserved core fold consisting of a seven strand β-barrel followed by an α-helix [11,15]. The PH domain of Sec7 has these structural elements too, however, when we modeled the Sec7 sequence to the crystal structure of the autoinhibited form of Grp1 Arf GTPase Exchange Factor we identified a nine strand β-barrel (Figure 1B). The signature motif for 3-phosphoinositide binding K X_m_ KxR X_n_ Y [11] is modified to I X_10_ SxK X_10_ F (m = 5-10; n = 6-13). Changes in these positions are also present in PH domains of other proteins (Figure 1C) [16]. A recently described glutamate in the PH domain of cytohesin-3 (GRP1), a sentry glutamate, appears to be essential for specific PI(3,4,5)P_3_ binding by the cytohesins as a charge reversal by mutation to lysine (E345K) enhanced the affinity for PI(4,5)P_2_ and yielded constitutive plasma membrane binding [17]. In Sec7 the glutamate is replaced by a glutamine which is considered a neutral residue (Figure 1A,B). A phylogenetic analysis of the Sec7 PH domain placed the *D. discoideum* protein close to GRP1 proteins of several species including the human protein (Additional file 1: Figure S1).

**Figure 2:**
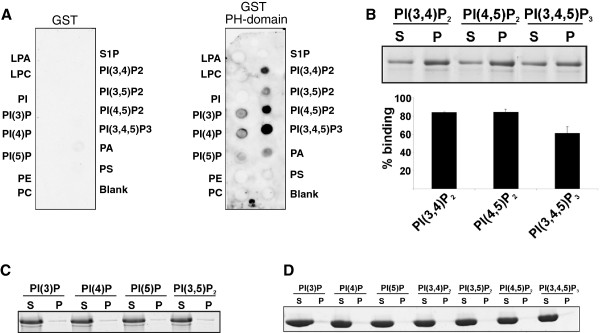
**The PH domain binds to phosphoinositides. (A)** PIP-Strip-membranes were incubated over night with GST-PH domain (1 μg/ml) and with GST (1 μg/ml) for control. Binding was detected by incubation with polyclonal GST-antibodies. **(B)** Binding of Sec7-PH domain (residues 454-577) to PIs in a liposome binding assay. Upper panel, 5 μg of the GST-PH fusion protein were incubated with liposomes containing 10% (wt/wt) of the indicated PIs. Liposomes were collected by centrifugation, and bound proteins resolved by SDS-PAGE and detected by Coomassie Blue staining (S = supernatant; P = pellet). Lower panel, for quantification, the bands were scanned and the ratio of the pellet vs. supernatant intensities of each PI fraction after background subtraction calculated. Error bars, SD of liposome binding from two independent experiments. **(C)** Binding of the Sec7 PH domain to PI(3)P, PI(4)P, PI(5)P and PI(3,5)P was negligible. **(D)** Liposome binding assay using GST for control.

We tested the ability of the PH domain of Sec7 to bind to different phosphoinositides in vitro and used a GST fusion protein encompassing residues 455-931 (GST-PH) in dot blot overlay assays. In this assay GST-PH bound preferentially to PI(3,4,5)P_3_ and showed decreased binding to PI(4,5)P_2_. GST alone did not show binding (Figure 2A). Although dot blot overlay assays are convenient assays, they need to be supported by different methods as apparent specificities can be distorted and as they do not allow reliable quantification [18]. We therefore carried out liposome binding assays in which we used liposomes containing 65% phosphatidylcholine, 20% phosphatidylethanolamine, 5% phosphatidylserine and 10% individual phosphoinositides and examined the sedimentation of GST-PH with the vesicles. The protein did not show significant binding to the monophosphorylated inositides PI(3)P, PI(4)P, PI(5)P and to the bisphosphorylated PI(3,5)P_2_, whereas it pelleted with PI(3,4)P_2_, PI(4,5)P_2_ and PI(3,4,5)P_2_. To quantitatively study which PIs are preferred, we scanned the band intensities of the Coomassie-stained gels and plotted the sedimented pellet fractions in a diagram. We found that in this assay the PH domain interacted equally well with PI(3,4)P_2_ and PI(4,5)P_2_ and exhibited less binding to PI(3,4,5)P_2_. The GST control showed no binding (Figure 2B,C,D).

A polybasic domain which follows the PH domains in all cytohesins is not present in Sec7. However, the C-terminus of Sec7 encompassing the PH domain and the remaining stretch of amino acids is highly basic with a pI of 9.58 which is presumably due to two poly asparagine stretches and an abundance of lysine residues near the C-terminus (10 lysine residues out of 56 residues).

**Figure 3:**
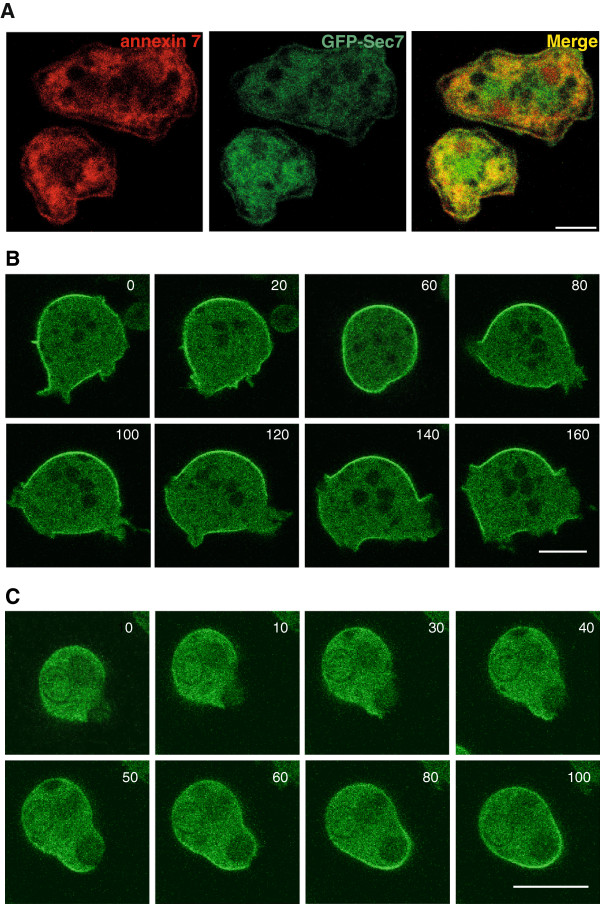
**Subcellular localization of GFP-Sec7.** To determine the localization of Sec7 we expressed a GFP-tagged Sec7 in Sec7 deficient cells. **(A)** GFP-Sec7 in methanol fixed cells. The cells were stained for annexin 7 with mAb 185-338-1 to reveal the plasma membrane. Annexin 7 is present in the cytosol, in the nucleus and at the plasma membrane. Bar, 5 μm. **(B)** Live cell analysis of GFP-Sec7 expressing cells. Representative images of a series are shown. Bar, 10 μm. **(C)** Live cell analysis of a GFP-Sec7 expressing cells during phagosome formation. Yeast particles were used to study phagocytosis. The time is given in seconds. Bar, 10 μm.

To address the localization of Sec7, we expressed the protein as a GFP fusion protein (GFP-Sec7) in a Sec7 deficient strain (see below). In fixed cells we found GFP-Sec7 diffusely distributed throughout the cytosol and present at the plasma membrane where it colocalized with annexin 7 [19] (Figure 3A). In live cell microscopy we observed GFP-Sec7 in association with the plasma membrane and in the cytosol. When cells extended pseudopods, GFP-Sec7 disappeared from the plasma membrane in the region of the newly forming protrusions (Figure 3B; 140, 160 sec). During macropinocytic cup formation, which allows cells to take up fluid [20], GFP-Sec7 was also excluded from the involved areas of the plasma membrane and reappeared upon completion of the process (Figure 3B, 80, 100 sec). Similarly, during phagocytosis of yeast particles GFP-Sec7 was not enriched at the forming phagocytic cup (Figure 3C; 10, 30, 40 sec). Once phagosome formation was completed and the yeast particle engulfed GFP-Sec7 reappeared on the plasma membrane (Figure 3C; 60, 80 sec). This behavior is unlike the one described for several PH-domains that recognize various phosphoinositides. It rather resembled the one of PTEN which is excluded from leading edges and whose distribution is complementary to the one of PI(3,4,5)P_3_[21]. The PH domain of PLCδ, which binds to PI(4,5)P_2_ and labels the plasmamembrane was similarly depleted from the active plasmamembrane [22]. Plasmamembrane regions of the cell involved in pseudopod extension, macropinosome and phagosome formation are highly dynamic and require membrane insertion as well as a dynamic underlying actin cytoskeleton which are processes triggered by transient PI(3,4,5)P_3_ formation. This analysis supports the proposed preference of Sec7 for PI(4,5)P_2_.

In cell fractionation experiments the protein was predominantly found in the cytosolic fraction which is in agreement with data obtained for cytohesins. A signal in the 100,000 × g pellet was only obtained when we loaded the material from 2×10^7^ cells/ml whereas for whole cell lysate and 10,000 × g pellet and supernatant, respectively, the proteins represent the material from 2×10^5^ cells/ml (Additional file 2: Figure S2A). We also expressed GFP-tagged Sec7 domain (residues 256-443) and found that it was present throughout the cytosol and did not relocate to the plasma membrane (data not shown).

### Identification of potential partners of Sec7

ARF GTPases are prominent interaction partners of the mammalian cytohesins. Further partners are components of cell adhesion complexes as cytohesin-1 for example interacts with the C-terminus of β2-integrin. Another component of focal adhesions, paxillin, was also found in a complex with cytohesins [23,24]. We carried out a preliminary analysis and used GST fusions encompassing the N-terminus (aa 1-382), the Sec7 domain (aa 256-443) and the C-terminus comprising the PH domain (aa 381-931) bound to glutathione sepharose beads, and antibodies against GFP for pull downs of potential binding partners in *sec7*^*-*^ expressing GFP-Sec7, AX2 and *sec7*^*-*^ expressing LimD-GFP for control. LimD is an unrelated protein which is present in the cytosol and near the plasma membrane and is well expressed as GFP fusion [25]. It did not show overlapping binding partners with Sec7. The proteins obtained were identified by mass spectrometry and classified as enzymes (11), cytoskeletal proteins (6), proteins involved in vesicle trafficking (14), in particular components of the endocytic machinery like NSF, the β-subunit of the AP1 complex which plays a role in clathrin-dependent protein sorting, delta adaptin belonging to the AP3 adaptor-like complex which participates in the generation of a diverse group of secretory organelles, and the gamma subunit of the coatomer complex essential for the secretory pathway, signaling proteins (4) and others (Table 1). Among the signaling proteins were zizA and zizB, two DOCK family proteins. Interestingly, for mammalian cytohesin-2 an association with Dock180 in a complex promoted ARF-to-Rac signaling [3]. When we used GO terms for classification, the majority of proteins from the pull downs fell into the category “transport” which was closely followed by “vesicle mediated transport”. Fewer proteins belonged to the category “biosynthetic process” and “small molecule metabolic process” (Additional file 3: Table S1). We confirmed the interaction for the cytoplasmic protein coronin and the 20S proteasomal subunit α-4, a further putative interaction partner, with antibodies in independent experiments (Additional file 2: Figure S2B, C).

**Table 1:** Potential binding partners of Sec7 and its domains

**Dictybase ID**	**Description**	**Identified in Sec7 domain pull down**	**Identified in PH domain pull down (C-terminus)**	**Identified in GFP-Sec7 immuno-precipitation**	**Identified in N-domain pull down**
**Membrane trafficking, membrane associated processes**					
DDB0237869	cog3, oligomeric Golgi complex component			+	
DDB0237794	osbH, oxysterol binding				+
DDB0185207	vatB, v-ATPase		+		
DDB0234266	vatH, v-ATPase		+		
DDB0233782	Present in macropinocytic proteome; conserved; transporter?		+		
DDB0185052	nsfA, N-ethylmaleimide-sensitive fusion protein		+		
DDB0234240	p3d1, delta adaptin (endosomal membrane)		+		
DDB0234067	Ap1b1, adaptor-related protein complex 1, beta 1 subunit, beta adaptin, highly similar to AP-1 complex subunit beta-1 (AP1B1) and AP-2 complex subunit beta-1 (AP2B1), which play a role in clathrin-dependent protein sorting	+			
DDB0304807	tgrO4, immunoglobulin E-set domain-containing protein, tgr (tiger) = Transmembrane, IPT, IG, E-set, Repeat protein	+			
DDB0233801	copG, adaptin N-terminal domain-containing protein	+			
	coatomer protein complex gamma subunit				
	gamma-COP				
DDB0306480	Single C-terminal TM	+			
DDB0238597	Dnajc13, DnaJ (Hsp40) homolog, subfamily C, member 13, very similar to the mammalian DnaJ homolog subfamily C member 13, required in D. melanogaster (Rme-8) for receptor-mediated endocytosis 8	+			
DDB0185049	myoI, class VII unconventional myosin	+			
	myosin VII, similar to the conserved MYO7A; unconventional myosin required in Dictyostelium for phagocytosis and substrate adhesion, interacts with talA				
DDB0234198	vps13A, vacuolar protein sorting-associated protein 13 family protein, putative ortholog of S. cerevisiae VPS13, involved in vacuolar protein sorting and protein-Golgi retention	+			
**Related to signal transduction**					
DDB0191258	pppA, protein phosphatase 2A subunit A		+		
DDB0235201	guanylate-binding protein	+			
	GTP-binding protein, 92 kDa				
DDB0233622	zizB, DOCK family protein, putative guanine nucleotide exchange factor (GEF)	+			
DDB0233623	zizA, DOCK family protein, putative guanine nucleotide exchange factor (GEF)	+			
**Cytoskeleton**					
DDB0201554	abpC, filamin		+	+	
DDB0233781	Centrosomal, 80 kDa		+		
DDB0191169	tubB, tubulin		+		
DDB0191115	corA	+	+	+	
DDB0185096	cytoplasmic dynein heavy chain,	+			
	dynein beta chain, flagellar outer arm				
DDB0185049	myoI class VII unconventional myosin	+			
	myosin VII, similar to the conserved MYO7A; unconventional myosin required in Dictyostelium for phagocytosis and substrate adhesion, interacts with talA				
DDB0191103	cortexillin I			+	

### Characterization of a *D. Discoideum* Sec7 deficient mutant

**Figure 4:**
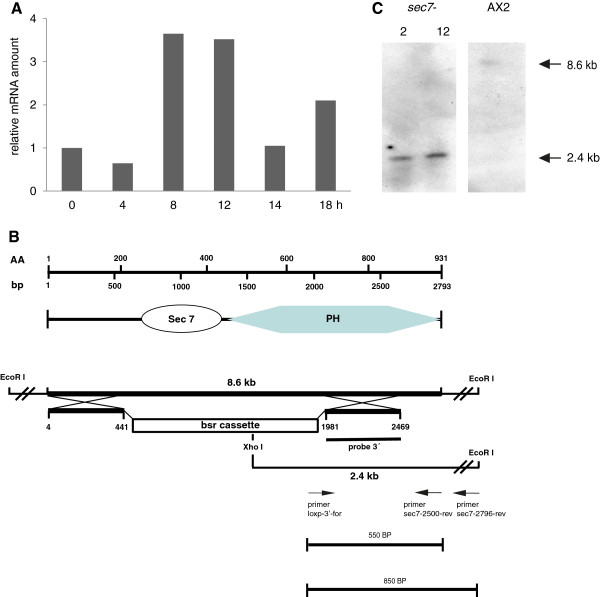
**Presence of Sec7 mRNA during *D. discoideum *development and generation of Sec7 deficient cells. (A)** Cells were developed on phosphate agar, mRNA was isolated at the indicated time points and RNA used for quantitative Real Time PCR. A 5’ fragment covering the first 500 bases of the cDNA was amplified. Relative values are shown. The mRNA content in growth phase cells (time 0) was arbitrarily set to 1.0 and taken as reference. For internal reference GAPDH amounts were determined. **(B)** Strategy used to generate the Sec7 gene replacement. From top to bottom: position of the nucleotides and the corresponding amino acids; the domain structure of the Sec7 protein; the genomic locus and the replacement vector. The location of the DNA probe used in the Southern blot analysis (3’ probe), the location of the PCR primers used for verification of the gene replacement event and the expected products are shown. **(C)** Southern blot analysis with DNA obtained from two independent clones and AX2 wild type. The DNA was digested with EcoRI and XhoI.

The sec7 gene of *D. discoideum* (DDB_G0272486) is located on chromosome 2. It contains one short intron of 86 nucleotides located in the first third of the gene (starting from position 947 of the cDNA sequence). *D. discoideum* cells enter a developmental program upon starvation. 8 to 12 hours after the start of starvation on phosphate agar plates cells form multicellular aggregates which transform into motile slugs at around 18 hours. Slugs are phototactic and thermotactic and finally form fruiting bodies. We followed the accumulation of the Sec7 mRNA during the life cycle and found that it is present during growth and development. The levels reach a maximum during the aggregation stage, drop after aggregation and increase again during the slug stage (Figure 4A).

For the generation of Sec7 deficient cells (*sec7*^*-*^) we used a gene replacement vector which contained nucleotides 1 to 441 and 1981 to 2469 of the cDNA. The intervening gene sequence was replaced by a blasticidin resistance cassette. Successful integration of the vector into the genome was confirmed by PCR and Southern blot analysis (Figure 4B,C). Several independent transformants were isolated, the characterization of one of them is shown. In the subsequent analysis we focused primarily on properties of the cells that are associated with processes involving regulation of the actin cytoskeleton and membrane dynamics.

**Figure 5:**
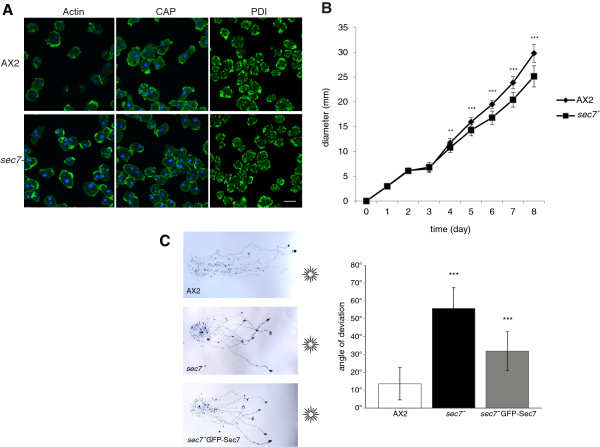
**Analysis of *sec7***^***-***^**cells. (A)** For immunofluorescence analysis AX2 and *sec7*^*-*^ cells were fixed with methanol and stained with mAb act1 to visualize the actin cytoskeleton, with mAb 223-451-1 to stain for actin associated CAP, and mAb 221-135-1 to visualize the ER marker PDI. Bar, 10 μm. **(B)** Growth on a lawn of *Klebsiella*. The growth of AX2 and mutant cells was followed over several days. The curves represent the measurements from more than one independent experiments in which six plates per strain were analyzed each. **(C)** Phototactic movement is altered in *sec7*^*-*^. In phototaxis experiments AX2 slugs migrate in a highly directed fashion towards the light source. At the end of the experiment they have reached the edge of the plate. *sec7*^-^ slugs migrate in a wider angle towards the light and have shorter trails (left panels). The behavior of *sec7*^*-*^ GFP-Sec7 slugs was improved. The position of the light source is indicated. The angle of deviation during slug phototaxis is significantly altered (bar graph on the right). The data are collected from between 6 and 10 experiments per strain. Each experiment included ~10 slugs. Unpaired *t* test was used for evaluation. P value is less than 0.0001 for *sec7*^*-*^, and for *sec7*^*-*^ GFP-Sec7 it is 0.0016.

Sec7 deficient cells were of the same size as the parent AX2 strain and were mainly mono- and binucleated. In immunofluorescence analysis we did not detect changes when we stained for F-actin, the actin-associated protein CAP and the endoplasmic reticulum located protein disulfide isomerase (PDI) as a marker for internal membranes (Figure 5A). Mutant cells grew well in shaking suspension with duplication times comparable to AX2 and reached similar final densities (~1×10^7^cells/ml). This is indicative of an unaltered macropinocytosis. Also, when grown on *E. coli* B/r as food source in shaking suspension, both *sec7*^*-*^ and AX2 had doubling times of ~3 hours suggesting that the uptake of *E. coli* was normal.

Growth on a lawn of *Klebsiella* as determined by measuring the increase in diameter of the colony was significantly reduced (Figure 5B). Such a behavior could be due to reduced phagocytosis or to altered motility. Hence we assayed the phagocytic capability following yeast particle uptake and found that fewer *sec7*^*-*^ cells had ingested one or more yeast particles after 45 min than AX2 cells. A quantitative evaluation showed that significantly fewer *sec7*^*-*^ cells (~79%) had taken up yeast cells as compared to AX2. *sec7*^*-*^ cells expressing GFP-Sec7 reached almost wild type level with ~95% (Additional file 4: Figure S3). The failure to completely restore wild-type behavior may lie in different expression levels and partial loss of expression.

Cell substrate and cell cell adhesion are two characteristics which are potentially influenced by the cytohesins as they affect events at the plasma membrane such as integrin activation [4]. Consequently we wanted to assess the role of Sec7 for these two aspects. Cell substrate adhesion is assayed by subjecting cells that have attached to a plastic surface to a rotation on an orbital shaker at 100 rpm. The percentage of detached cells after one hour was similar for AX2 and mutant cells. Upon increase of rotation to 160 rpm the results for wild type and mutant showed no significant difference either (data not shown). Cell cell adhesion was tested in developing cells. During development *D. discoideum* cells express specific cell surface proteins like contact site A which mediate cell adhesion and allow aggregate formation [26]. When we assayed aggregate formation during development in shaking suspension by following the decrease in optical density of the suspension we did not detect differences between wild type and mutant (data not shown). This was also taken as an indication for correct expression of the cell adhesion molecules and for normal development of the mutant.

Later developmental stages were assayed by depositing cells on phosphate agar plates for starvation. The *sec7*^*-*^ strains formed aggregates, mounds, slugs and fruiting bodies in a timely fashion. Furthermore, the fruiting bodies had comparable morphologies (data not shown).

Slugs are phototactic and migrate towards light. Phototaxis is an essential feature in the wild where *Dictyostelium* has to reach the soil surface from where the spores can be dispersed. Directed migration of slugs towards light was tested by keeping the plates in the dark and providing a lateral light source. After incubation for two days the trails of the slugs were stained with Amido Black and the migration pattern and the light sensing evaluated. AX2 slugs migrated over long distances and almost directly towards the light source. Many of the slugs had reached the edge of the plate. *sec7*^*-*^ slugs had a phototaxis defect. Their migration trails were shorter and the slugs never reached the edge of the plate. Moreover, their directionality was altered and the angle of deviation during slug phototaxis was increased. AX2 slugs migrate with an angle of ~13 degrees, *sec7*^*-*^ slugs with an angle of ~55 degrees and for *sec7*^*-*^ slugs expressing Sec7-GFP the angle was reduced to ~31 degrees (Figure 5C). We conclude that *sec7*^*-*^ slugs can sense light, but directionality is impaired.

As we had identified components associated with vesicle trafficking in our co-precipitation experiments with individual domains of Sec7 and the full length protein we tested the secretion of a lysosomal hydrolase and of cAMP phosphodiesterase. Lysosomal α-mannosidase is produced during growth and the first hours of development. The protein undergoes a variety of posttranslational modifications during its transit from the ER and Golgi to the late lysosomes before it is secreted [27]. We tested AX2 wild-type and *sec7*^*-*^ cells for the ability to secrete α-mannosidase during the first hours of development. ~60 and 64% of the total enzyme activity were found in the medium of AX2 and *sec7*^*-*^ cells, respectively, after 2 hours of development in shaking suspension. After 6 hours the values reached 75 and 77% of total enzyme activity. We concluded that secretion occurred normally in *sec7*^*-*^, however, there was consistently less active enzyme present amounting to ~76% of wild-type activity (Tables 2 and 3). Secretion of cAMP phosphodiesterase was measured during early development by measuring phosphodiesterase activity in the medium. We observed a linear increase of activity in all strains, however, *sec7*^*-*^ had secreted less enzyme and the amounts were reduced to ~50% of wild-type level. Form the analysis of the data it appears that the mutant produces less enzyme and that secretion is affected as well as. GFP-Sec7 showed an improvement reaching 60% of AX2 levels (Table 4).

**Table 2:** **Ability of *sec7***^***- ***^**cells to secrete enzymes during early development: secretion of α-Mannosidase**

**Strain**	**% secreted enzyme after**		**% total enzyme activity after 6 hours**
	**2 hours**	**6 hours**	
AX2	60	75	100
*sec7*^*-*^	64	77.4	75.80

Cells were starved in Soerensen phosphate buffer, pH 6.0, and at the indicated time points the α-mannosidase activity was determined. α-mannosidase secretion in percent and total α-mannosidase activity in percent are shown. The α-mannosidase activity was determined in the medium and in the cell pellet at the indicated time points after the beginning of starvation. Values are from one representative experiment.

**Table 3:** **Ability of *sec7***^***- ***^**cells to secrete enzymes during early development: total mannosidase activity in wild type and mutant cells at various stages of development**

**Strain**	**AX2**			***sec7***^***-***^		
	**t0**	**t4**	**t6**	**t0**	**t4**	**t6**
	3.72	4.42	4.26	2.3	2.58	2.67
	2.58	3.33	3.65	1.84	2.22	2.64
	1.5	2.38	2.2	1.29	2.19	2.24
	3.25	3.6	3.8	1.393	2.03	2.37
	3.73	4.12	4.44	0.74	1.05	1.25
SD	0.93948	3.5700	0.8831	1.51260	0.5752	0.5792
SEM	0.42015	0.3535	0.3949	0.58906	0.2572	0.2590
Mean	2.95600	3.5700	3.6700	0.26343	2.0140	2.2340
P value				0.0196	0.0074	0.0160

Time points (t) are given in hours. Five experiments were analyzed. For statistical analysis an unpaired t test was used. The differences were statistically significant.

**Table 4:** **Ability of *sec7***^***- ***^**cells to secrete enzymes during early development: phosphodiesterase production**

	**PDE activity (U/ml) determined at the indicated time points after begin of starvation**					
	**AX2 (t1)**	***sec7***^*- *^**(t1)**	**AX2 (t2)**	***sec7***^*- *^**(t2)**	**AX2 (t3)**	***sec7***^*- *^**(t3)**
Mean	2.3767	1.1600	13.3567	6.0700	26.8433	12.6467
SD	0.6486	0.6161	2.9151	2.3226	2.0385	1.8360
SEM	0.3744	0.3557	1.6831	1.3410	1.1769	1.0600
N	3	3	3	3	3	3
P value		0.0780		0.0276		0.0034

Mutant and wild type cells were starved in Soerensen phosphate buffer, pH 6.0. At the indicated time points in hours after begin of starvation the activity of extracellular phosphodiesterase (in units/ml) was determined. The results from three experiments were evaluated. N, number of experiments. The difference in secretion obtained for *sec7*^*-*^ at 2 and 3 hours was statistically significant.

### Analysis of signaling to the actin cytoskeleton, cell motility and chemotactic behaviour

**Figure 6:**
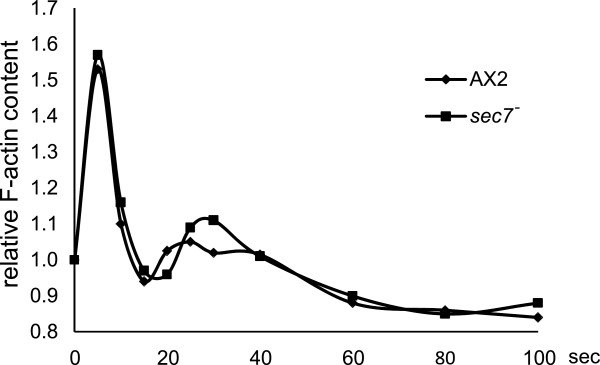
**F-actin accumulation after a cAMP stimulus.** cAMP-induced F-actin formation as measured with a phalloidin binding assay. Aggregation competent AX2 wild-type and *sec7*^*-*^ cells were stimulated with cAMP and the F-actin content was determined at the indicated time points. Results from a representative experiment are shown.

On application of cAMP to cells, actin polymerization and depolymerization occurs in a characteristic pattern. After an initial increase of the F-actin concentration, the filaments depolymerize quickly. This phase is followed by a long lasting phase of F-actin polymerization paralleling the formation of pseudopods. We tested whether Sec7 plays a role in the control of dynamic actin rearrangements and examined the actin polymerization response to cAMP stimulation. We found that in *sec7*^*-*^ the relative F-actin content as revealed by TRITC-phalloidin staining of samples taken at different time points exhibited the typical biphasic pattern with peaks at 5 and 30 seconds as in AX2 suggesting that Sec7 is not required for cAMP-induced actin polymerization (Figure 6).

*Dictyostelium* cells exhibit an amoeboid type of cell motility. To assess the role of Sec7 for motility we analyzed the motility of single cells during the aggregation phase after six hours of starvation. Both AX2 and *sec7*^*-*^ cells were highly polarized and migrated towards a cAMP source, but the speed of the *sec7*^*-*^ cells was significantly reduced as compared to AX2. AX2 cells migrated with a speed of 14.22±2.00 μm/min, *sec7*^*-*^ cells with 8.66±2.25 μm/min. The direction change reflecting the frequency of turning was similar, however, persistence which indicates the probability to continue movement in the same direction was significantly lower (Table 5).

**Table 5:** Cell motility of aggregation stage cells

	**Speed (μm/min)**	**Persistence (μm/min-deg)**	**Direction change (deg)**	**n**
AX2	14.22±1.99	5.47±1.58	14.69±4.88	29
*sec7*^*-*^	8.66±2.25	3.3±1.33	16.28±6.84	31
P value	0.0001	0.0001	NS	

Persistence is a measure for the probability to continue movement in the same direction. The direction change (deg/min) reflects the frequency of turning. n, number of cells analyzed. The difference in speed and persistence was statistically significant. Cells from four independent experiments were evaluated. NS, not significant.

## Discussion

The cytohesin family of GEFs are activators for ARF GTPases. They have a broad range of roles ranging from signal transduction to cytoskeletal organization which can occur in an ARF-dependent and an ARF-independent function. Our analysis of Sec7 deficient *D. discoideum* cells revealed that loss of the protein affected several cellular processes including altered secretion, growth under certain conditions, phagocytosis, cell motility and phototaxis (Table 6). These processes are complex and several of them involve membrane flow and the actin cytoskeleton and the individual contributions are not easily deciphered. One example is phagocytosis of large particles like yeast cells which was significantly impaired whereas uptake of *E. coli* B/r was as efficient as in AX2 wild type cells. Such a differential behavior might be explained by earlier findings showing that *D. discoideum* can differentiate particles according to their surface properties and geometry. Furthermore, analysis of the actin organization around the phagosome revealed a distinct and dynamic spatiotemporal pattern [22,28]. A role of Sec7 in phagocytosis is also supported by the potential binding partners as several of them have been implicated in this process and found associated with the phagosome [29]. We also noted changes when we tested the secretion of α-mannosidase and cAMP phosphodiesterase. Both enzymes are secreted during early development. In case of α-mannosidase we observed a similar rate of secretion as in AX2, for cAMP phosphodiesterase it was reduced. The total enzyme levels were significantly reduced for both. The second major defect was in cell migration where the *sec7*^*-*^ cells were slower and less persistent. For directed cell motility and chemotaxis phospholipid signaling and lipid metabolism is essential. In particular, PI(3,4,5)P_3_ controls cell speed and pseudopod formation [30]. The reduced cell speed and less persistent migration of the *sec7*^*-*^ cells parallels the behavior of PTEN deficient *D. discoideum* cells with which Sec7 shares the constitutive localization at the plasma membrane and an absence from newly forming pseudopods. This might suggest that Sec7 acts downstream of PTEN [31].

**Table 6:** **Summary of phenotypes observed in the *sec7***^***- ***^**strain**

**Phenotype**	***sec7***^***-***^
Cell size	wt
Nuclei number	wt
Growth in axenic medium (measure of macropinocytosis)	wt
Growth on *E. coli* in shaken suspension	wt
Growth on a lawn of Klebsiella	reduced
Phagocytosis of yeast cells	uptake reduced to 60% of AX2 level
Development	wt
Cell substrate adhesion	wt
Cell cell adhesion	wt
Phototaxis	directionality impaired, slug trail length reduced
Localization of F-actin and actin associated proteins (actin, CAP)	wt
Integrity of ER membrane system (PDI staining)	wt
Secretion of α-mannosidase	secretion occurs normally, however enzyme levels are reduced (~76% of AX2 level)
Secretion of phosphodiesterase	secretion appears to be impaired, enzyme levels are reduced (~50% of AX2 level)
F-actin assembly after cAMP stimulation	wt
Cell motility	speed reduced
Persistence during migration	lower

**“**wt” describes wild type behavior.

Members of the cytohesin family of GEFs are recruited to the plasma membrane based on the affinity of their PH domain for specific phosphatidylinositol phosphates. A further mechanism is through interaction with the GTP bound form of ARF6 and ARL4 which leads to cytohesin recruitment and activation of ARF6 and ARF1 [32-34]. The activation of ARF proteins stimulates signaling pathways that regulate membrane trafficking and cell motility. Mammals have six ARFs forming three classes and playing roles in different cellular pathways [35]. *D. discoideum* harbors a single ARF homolog encoded by the arfA gene on chromosome 5. This is similar to *Giardia lamblia*, a flagellated protozoan parasite, which also has one ARF only. ArfA (DDB0191101) is most closely related to ARF1 with 85% identical and 90% homologous residues when compared to human ARF1 (NP_001019397.1) (E value, 3e-86) and less related to human ARF6 (CAG46762.1) sharing 67% identical and 82% homologous residues (E value 1e-69). In addition to ArfA *D. discoideum* has ten ARF related proteins, ArrA–K. ArfA is present in a macropinocytic proteome [36] whereas this has not been reported for Sec7. ArfA has also been studied by Chen et al. [37] who showed that two ArfGAPs belonging to the ASAP/ACAP type of ArfGTPases, which are distinguished by BAR, PH and Ank domains, act on ArfA. The *D. discoideum* ArfGTPases have roles in actin cytoskeleton organization and spore production. The studies on ACAP were more recently extended by Dias et al. [38] who revealed additional roles in cytokinesis, cell migration and cytoskeleton dynamics.

Another ArfA binding protein in *D. discoideum* is AdcA. This arrestin related protein bound ArfA in its GDP-bound conformation [39]. AdcA was associated with the endocytic pathway and early endosomes, and faintly labeled the plasma membrane. GFP-tagged ArfA localized in the cytosol, at the plasma membrane and mainly in the perinuclear region at the Golgi apparatus. From in vivo studies the authors concluded that ArfA-GFP was present on vesicles and tubules moving towards and away from the Golgi apparatus. They proposed a role for ArfA in trafficking events that link the Golgi to other organelles such as endosomes. Although ArfA had been found in proteomic studies on phagosomes and macropinosomes [36,40], ArfA-GFP was not convincingly located at these structures in vivo which was thought to be due to transient interactions. The absence of GFP-Sec7 from these locations in our studies is compatible with these results.

In our attempt to identify potential binding partners of Sec7 we carried out pull downs using domains of Sec7 expressed as GST fusion proteins in *E. coli* and full length protein expressed as GFP fusion in *D. discoideum*. The majority of the putative interaction partners from all pull downs belonged to the processes of trafficking and vesicle trafficking although the overlap between the individual pull downs was limited. This could be due to the conformations of the proteins and the different accessibilities of the sequences. For some of the putative interaction partners mutants are available and their phenotypes can be compared to the one of the *sec7*^*-*^ mutant. We did see overlaps of the phenotypes in several cases which might indicate that the proteins act in the same pathway. This applies particularly to phagocytosis and chemotactic motility (Additional file 3: Table S2).

Phosphoinositides are tightly regulated during chemotaxis in *D. discoideum*, in particular, PI(3,4,5)P_3_ gradients are formed within the plasma membrane. They are thought to be of differing importance for sensing of shallow and steep cAMP gradients [30,41]. The PH domain of the cytohesin family of ARF-GEFs can act as PI(3,4,5)P_3_ sensor. We found that *D. discoideum* Sec7 had highest affinity for this phosphoinositide in lipid overlay assays followed by PI(4,5)P_2_, whereas in liposome binding assays it preferred PI(4,5)P_2_ and PI(3,4)P_2_ over PI(3,4,5)P_3_. When we analyzed GFP-tagged Sec7 in vivo the protein decorated the plasma membrane and it did not visibly associate with those membrane regions where PI(3,4,5)P_3_ formation is thought to occur. The group of C. Weijer [21] had used a panel of PH domains with specificities for PI(3,4,5)P_3_ and PI(3,4)P_2,_ among them the PI(3,4,5)P_3_ specific PH domain of GRP1 (cytohesin-3), with which they analyzed the formation of these signaling molecules during phagocytosis and chemotaxis. They concluded that PI(3,4,5)P_3_ levels transiently increased during phagocytosis and macropinosome formation at sites of engulfment as revealed by the recruitment of GRP1-PH to these regions. During chemotaxis towards cAMP PI(3,4,5)P_3_ was formed and degraded to PI(4,5)P_2_ in the plasma membrane. GRP1-PH and CRAC-PH, a *D. discoideum* protein specific for PI(3,4,5)P_3,_ translocated to the plasma membrane following cAMP stimulation [42]. Interestingly, the Weijer group found that different PI(3,4,5)P_3_ binding PH domains behaved differently as GRP1-PH exhibited maximum binding several seconds later than other PH domains and remained at the membrane much longer which might be indicative of further determinants.

GFP-Sec7 did not exhibit a comparable pattern of plasma membrane association. Instead, it was constitutively associated with the plasma membrane and consistently disappeared from regions forming a new pseudopod, undergoing phagocytosis or macropinocytosis which require the insertion of new membrane. Sec7-GFP showed a behavior complementary to the one of PH domains sensing PI(3,4,5)P_3_ and rather resembled the pattern reported for the phosphatase PTEN and the PH domain of PLCδ [43]. Like Sec7 deficient cells the PTEN null cells showed an impairment in phagocytosis of yeast cells and a normal uptake of bacteria [20]. They also had a cell migration defect [43,44].

## Conclusion

The analysis of a *Dictyostelium* Sec7 mutant implicates the protein in processes that are related to membrane flow and actin dynamics and reveals a conserved function for this class of proteins. Its PH domain has the potential to recognize PI(4,5)P_2_ and to a lesser extent PI(3,4,5)P_3_ in vitro. The protein associates with the plasma membrane, however, during macropinocytosis, phagocytosis and pseudopod extension it is lost. It therefore does not act as a sensor for 3-phosphoinositide dynamics. This is supported by the absence of the sentry glutamate in Sec7 which in the cytohesins is essential for specific plasma membrane targeting. Instead, the glutamate is replaced by glutamine. The Sec7 plasma membrane association is presumably mediated by PI(4,5)P_2_ binding, however, other determinants such as binding to interacting proteins might also be important factors.

## Materials and methods

### Growth and development of *D. Discoideum* strains and mutant generation

*D. discoideum* strains used were AX2, a Sec7 deficient strain derived from AX2 (*sec7*^*-*^), and *sec7*^*-*^ expressing GFP-Sec7. They were grown in shaking suspension (160 rpm) in axenic medium at 22ºC or on a lawn of *Klebsiella* on SM agar plates [44]. For growth on *E. coli* in shaking suspension (160 rpm) *E. coli B/r* was harvested and resuspended in Soerensen phosphate buffer (17 mM sodium-potassium-phosphate, pH 6.0) at a density of 10^10^ cells/ml. Inoculation was with 5×10^5^*D. discoideum* cells/ml. Growth was determined by following the increase in cell number over time. Development was done with cells starved in Soerensen phosphate buffer at a density of 1×10^7^ cells/ml in shaking suspension. Under these conditions, development proceeded until the tight aggregate stage. Upon development on a solid substratum fruiting body formation occurred. For this 5×10^7^ cells were spread onto phosphate agar plates (10 cm in diameter) and incubated at 22ºC until fruiting bodies had formed. For evaluation of slug migration and phototaxis 5×10^5^ cells in 5 μl Soerensen phosphate buffer were spotted in the center of a water agar plate. Incubation was in the dark with a lateral light source. After 48 hours cells had formed slugs which migrated towards the light. They were transferred to nitrocellulose filters and detected by staining with Amido Black (0.1% in 25% isopropanol and 10% acidic acid).

The Sec7 cDNA was amplified from cDNA that had been prepared from strain AX2, cloned into pGEM-T Easy (Promega). The sequence was verified and used for all further cloning steps. For inactivation of the Sec7 gene, a gene replacement vector was generated. Residues 4 to 441 and 1981 to 2469 of the cDNA were cloned into pGEM-T Easy carrying a Blasticidin resistance gene under the control of the actin 15 promoter [45]. The plasmid was transformed into AX2 and transformants were selected using Blasticidin S (MP Biomedicals, Eschwege, Germany) at 1.5 μg/ml. Single colonies were selected on a *Klebsiella* lawn, DNA was isolated from nuclei using phenol/chloroform extraction [44] and PCR analysis was carried out with primers that revealed the gene replacement event. The gene replacement was further confirmed by Southern blot analysis. Several independent clones were identified, two of them were further analysed. As the results did not differ, the characterization of one of the clones is presented in the Results section.

### Expression of recombinant protein

For expression of recombinant Sec7 polypeptides as glutathione S transferase (GST) fusion proteins in *E. coli*, cDNA fragments encoding the N-terminal domain (amino acid residues 1-382), the Sec7 domain (amino acid residues 256-443) and the C-terminus encompassing the PH domain (amino acid residues 381-931) and a polypeptide containing only the PH domain (amino acid residues 454-577) were cloned into pGEX vectors (GE Healthcare Life Sciences). *E. coli* strains XL1 Blue and, in case of the Sec7 domain, Arctic Express Ril (Agilent Technologies) were used for expression of the GST fusion proteins. Full length Sec7 cDNA was cloned into pDex79 and expressed as GFP fusion (GFP-Sec7) under control of the actin 15 promoter [46]. GFP was fused to the N-terminus of Sec7. The plasmid was transformed into *sec7*^*-*^ cells. Selection was with G418 (Life Technologies Corporation) at 4 μg/ml. For expression of the Sec7 domain, cDNA sequences corresponding to amino acid residues 252–443 were cloned into pDex79.

### Pull down assays and immunoprecipitation

To identify interaction partners of Sec7, GST-Sec7 N-terminus, GST-Sec7 domain, GST-Sec7 C-terminus and GST for control were bound to glutathione sepharose 4B beads (GE Healthcare) and used for pull down assays. Incubation with cell lysates varied from 2 hours to overnight and was performed at 4ºC. Lysates were prepared from AX2 cells, *sec7*^*-*^ expressing Sec7-GFP and *sec7*^*-*^ cells expressing LimD-GFP [25] using the following buffer: 10 mM Tris-HCl, pH 7.5, 150 mM NaCl, 1% NP 40, 0.5 mM EDTA, 1 mM PMSF and protease inhibitors (Sigma). Lysis was controlled microscopically. For immunoprecipitation of GFP-Sec7, monoclonal antibodies (mAb K3-184-2) as well as polyclonal antibodies against GFP bound to protein A sepharose beads were used [47]. Protein A sepharose beads were used as control to exclude proteins that bind to the sepharose matrix. The proteins from pull downs, immunoprecipitations and controls were separated by SDS-PAGE (10% to 12% acrylamide), stained with Coomassie Blue, bands from control and experiment were cut out and proteins processed for LC-MS at the Bioanalytics Facility of the CMMC. The Mascot search engine was used for identification of the proteins. For verification of the interaction immunoprecipitation of GFP-Sec7 was repeated and probed for the presence of coronin and subunit α-4 (psmA4, (DDB0214953)) of the 20S proteasome using mAb 176-3-6 and 159-83-10, respectively [48,49].

### Mutant analysis

Growth analysis, uptake of yeast particles and adhesion assays were done as described [5], analysis of F-actin assembly after cAMP stimulation was done as described [47]. Mannosidase activity in cell pellets and in the supernatant was determined according to Loomis [50]. In brief, cells were starved at a density of 1×10^7^ cells/ml. At the beginning of the experiment (t0) and after 2, 4 and 6 hours 500 μl were taken to measure mannosidase activity. For determination of secreted enzyme 100 μl of the supernatant were mixed with 100 μl Na-citrate buffer (pH 5.0) and 200 μl substrate solution (2 μl p-nitrophenyl-α-D mannopyranoside (150 mM). The substrate was dissolved in DMF. The reaction was stopped after 30 min incubation at 37ºC by addition of 600 μl sodium borate (0.2 M, pH 9.8) and the product extracted into butanol. Nitrophenol formation was estimated by measuring the absorbance at 405 nm. For determination of total enzyme activity cells were lysed by addition of Triton X-100 (0.5%). cAMP phosphodiesterase (PDE) activity was determined using a coupled enzymatic assay with cAMP as substrate [51]. AMP, the product of the hydrolysis, was further converted to IMP by adenosine deaminase. IMP was then converted to inosine by alkaline phosphatase. The decrease of the absorption at 265 nm was a measure of inosine formation which in contrast to adenosine does not absorb light at 265 nm. Reagents and enzymes were from Sigma. For immunofluorescence analysis methanol fixed cells were stained for actin (mAb act1 [52]), CAP (mAb 223–445-1 [53]), protein disulfide isomerase (PDI, mAb 221-135-1 [54]) and annexin 7 (mAb 185-338-1 [19]). For detection goat anti mouse antibodies coupled to Alexa Fluor 488 (Life technologies) were used. Analysis of fixed and living cells was done by laser scanning confocal microscopy using a Leica TCS SP5 microscope.

For cell motility analysis cells were plated after ~ 6 hours of starvation in a chamber (ibidi GmbH-Martinsried, Germany) and migration towards aggregation centers or towards a micropipette filled with 10 μm cAMP was followed. Analysis was carried out by using the DIAS system as described [5]. For phototaxis 5×10^5^ cells are placed in the center of a water agar plate. Slugs were allowed to form and migrate towards light. After 48 h, slugs and slime trails were transferred to nitrocellulose filters and stained with Amido Black.

### Liposome binding assay

Phosphatidylserine (PS), phosphatidylcholine (PC), phosphatidylethanolamine (PE), PI(3)P, PI(4)P, PI(5)P, PI(3,4)P2, PI(3,5)P2, PI(4,5)P2, and PI(3,4,5)P3 were obtained from Sigma and diluted in chloroform. Liposome binding experiments were performed with a modified published liposome binding assay protocol [55]. Lipid mixtures containing 65% PC, 20% PE, 5% PS and 10% individual phosphoinositides were produced by mixing appropriate lipid solutions in chloroform/methanol. Slow flow nitrogen gas was used for the production of a film on the glass and vacuum desiccation for 30 min for solvent removal. Sterile-filtered sucrose binding buffer (20 mM HEPES, pH 7.4, 100 mM KCl, 1 mM EDTA and 0.1 M sucrose) was added to a final lipid concentration of 1mg/ml and incubated at 37ºC for 2 hrs. Lipids were then sonicated in a waterbath-sonicator for 10 sec.

To test liposome binding, a 100 μl reaction mixture of freshly prepared liposomes and 5 μg of purified protein were incubated for 15 min at room temperature and centrifuged at 100,000 × g (42,000 rpm) at 4ºC for 25 min in a Beckman table top ultracentrifuge Optima TLX (TLA 45 rotor). The supernatant was saved, and the pellet was resuspended in 100 μl of sucrose binding buffer. Both fractions were then analyzed by SDS-PAGE followed by Coomassie blue staining. ImageJ was used for quantification.

### Miscellaneous methods

Phosphoinositide-binding assays using lipid strips supplied by Echelon Biosciences, Inc. (Salt Lake City, Utah, USA) were performed as described [56]. For statistical analysis the Student's *t* test was used. For cell fractionation cells were lysed using Nuclepore filters (Whatman) in 20 mM Tris-HCl, pH 8.0, 50 mM NaCl and protease inhibitors (Sigma). Sequential centrifugation steps were done at 400 × g (2 min) to remove unlysed cells, 10,000 × g (10 min) to pellet nuclei and 100,000 × g (60 min) to separate membrane and cytosolic fractions. Proteins were separated on SDS-PA gels (10% acrylamide), blotted onto nitrocellulose membranes and GFP-tagged protein detected with mAb K3-184-2 and for a cytosolic control protein using enhanced chemiluminescence [47]. Total RNA was isolated using phenol extraction. Quantitative Real Time PCR was done as described [57].

## Competing interests

The authors declare that they have no competing interests.

## Authors’ contributions

AAN, TJR, WK and MS conceived the project, RM, CH, SKS, NNO, MP, TJR, MS, KS, MT, KY, RB-W, and CG designed and performed the experiments and analyzed the data. AAN, TJR, WK and MS analyzed the data and wrote the manuscript. All authors read and approved the final manuscript.

## Supplementary Material

Additional file 1: Figure S1 Evolutionary tree of the Sec7-PH domains. CLUSTALX alignments of the Sec7-PH domains from *Homo sapiens* and *Dictyostelium discoideum* and other selected organisms were used to create dendograms with TreeView. Boot strap values are provided at the node of each branch. The scale bar indicates amino acid substitutions per site. Organisms used: hs: *Homo sapiens*, ce: *Caenorhabditis elegans,* dd: *Dictyostelium discoideum,* dm: *Drosophila melanogaster,* sp: *Schizosaccharomyces pombe,* mo: *Magnaporthe oryzae,* as: *Arabidopsis thaliana, Os: Oryza sativa.* Click here for file

Additional file 2: Figure S2 **(A)** Cell fractionation reveals the presence of GFP-Sec7 in the cytosol and in membrane fractions. The cells were opened using Nuclepore filters and aliquots separated by SDS PAGE (10% acrylamide). Proteins were detected with mAb K3-184-2 [47]. L, whole cell lysate, P0 (400 × g); SN1, P1 (10,000 × g); SN2, P2 (100.00 × g). SN, supernatant; P, pellet. The signal obtained for whole cell lysate, 10,000 × g pellet and supernatant and 100,000 × g supernatant represents the material from 2×10^5^ cells/ml, the signal in the 100,000 × g pellet corresponds to 2×10^7^ cells/ml. **(B)** Coronin and proteasomal subunit interact with GFP-Sec7. GFP-Sec7 was immunoprecipiated using GFP antibodies and the pull down probed for the presence of coronin using mAb 176-3-6 and mAb 159-83-10 to detect the 28 kDa proteasomal subunit psmA4 (DDB0214953). psmA4 was observed in one pull down experiment only and was therefore not included in Table 1. Here it proved to be a binding partner. For control, *sec7*^*-*^ expressing GFP-LimD was used. **(C)** Coimmunoprecipitation of GFP-Sec7 with coronin. Protein A sepharose beads carrying mAb 176-3-6 were used to precipitate coronin from cell lysates of *sec7*^*-*^ cells expressing GFP-Sec7 or GFP-LimD and the immunoprecipitates probed for the presence of GFP-Sec7 using GFP-specific antibodies. Click here for file

Additional file 3: Table S1 GO classification of the Sec7 interaction partners and **Table S2** Mutant phenotypes of potential Sec7 interaction partners. The GO analysis was carried out at http://go.princeton.edu/cgi-bin/GOTermMapper. Click here for file

Additional file 4: Figure S3 *sec7*^*-*^ cells have a phagocytosis defect. AX2, *sec7*^*-*^ and *sec7*^*-*^ expressing GFP-Sec7 cells were incubated with yeast and fixed after 45 minutes. The number of cells containing yeast particles was determined. The results shown are from three independent experiments. P values are given. The difference between *sec7*^*-*^ and AX2 is significant. Click here for file
