# Supplementary figures and images for: The cytohesin paralog Sec7 of Dictyostelium discoideum is required for phagocytosis and cell motility
Source: Cell Commun Signal. 2013 Aug 1;11:54. doi: 10.1186/1478-811X-11-54 (PMC3737031; doi:10.1186/1478-811X-11-54)

## Slide 1
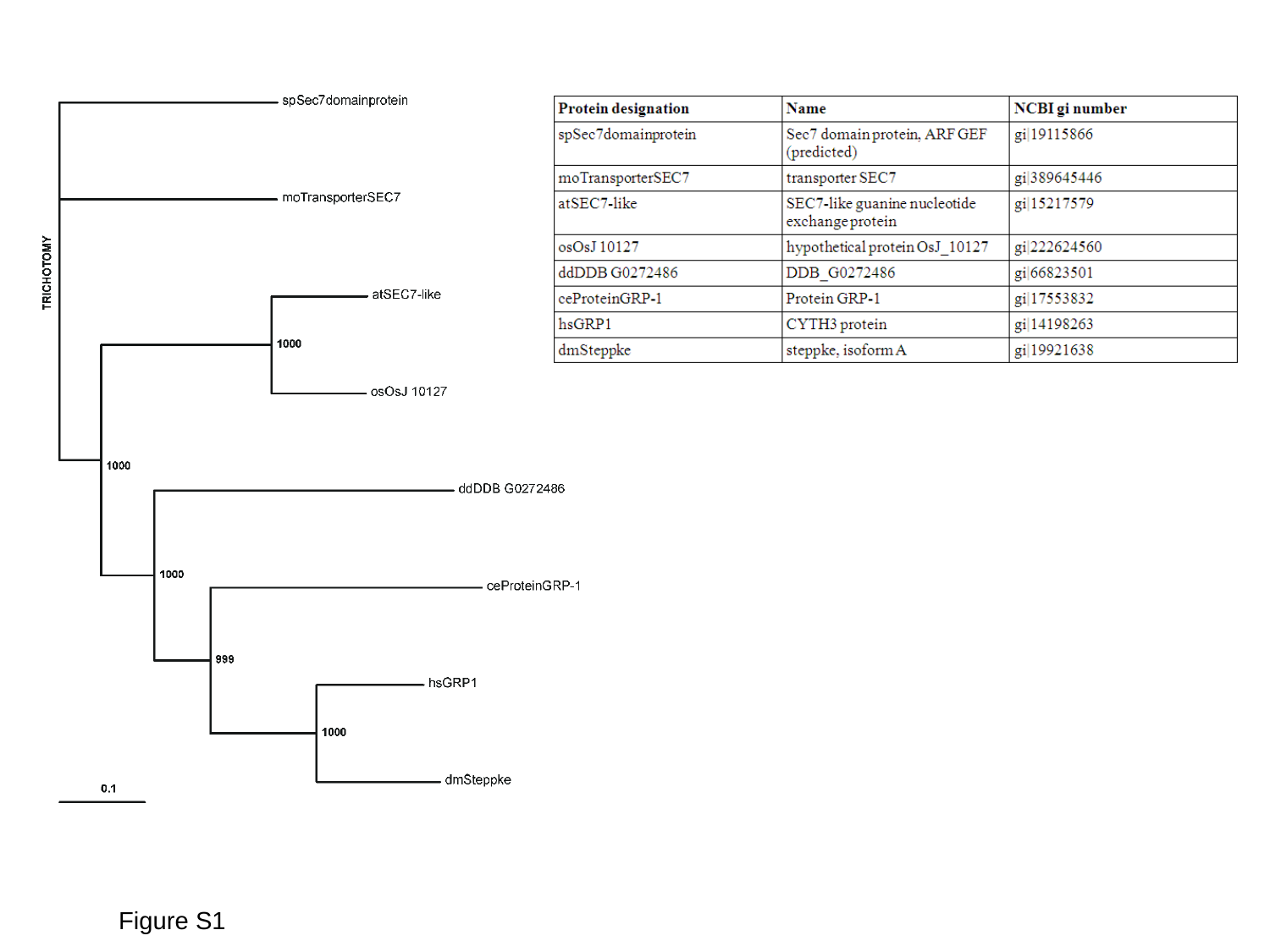

Figure S1

Supplement: Additional file 1: Figure S1 — Evolutionary tree of the Sec7-PH domains. CLUSTALX alignments of the Sec7-PH domains from Homo sapiens and Dictyostelium discoideum and other selected organisms were used to create dendograms with TreeView. Boot strap values are provided at the node of each branch. The scale bar indicates amino acid substitutions per site. Organisms used: hs: Homo sapiens, ce: Caenorhabditis elegans, dd: Dictyostelium discoideum, dm: Drosophila melanogaster, sp: Schizosaccharomyces pombe, mo: Magnaporthe oryzae, as: Arabidopsis thaliana, Os: Oryza sativa. [file 1478-811X-11-54-S1.pptx]

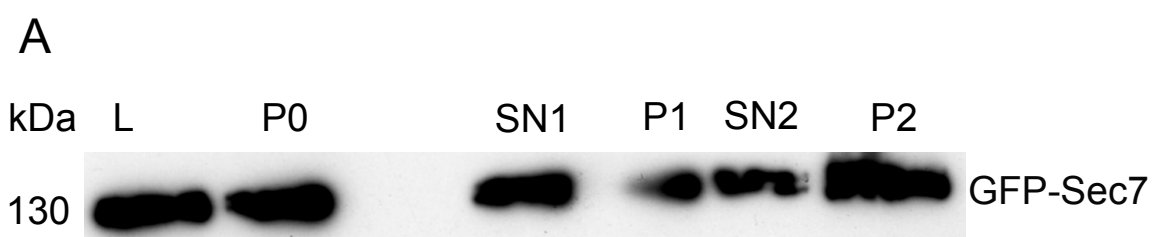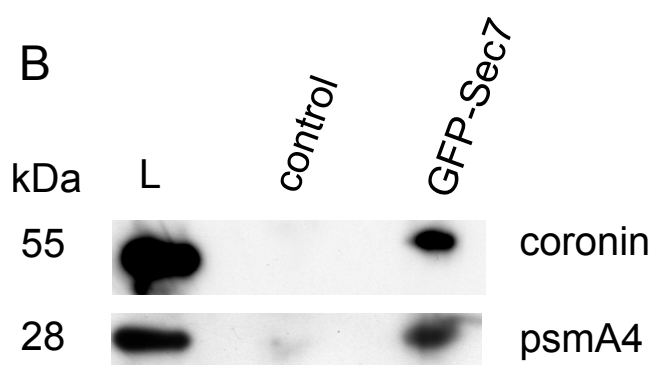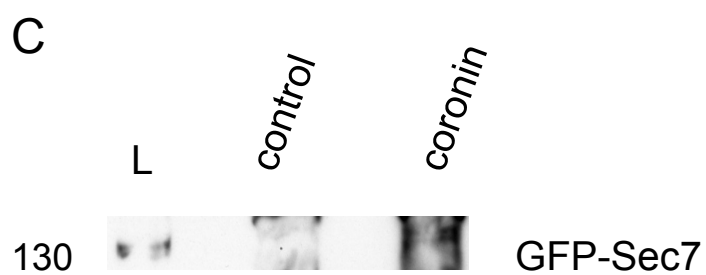

Figure S2A,B,C

Supplement: Additional file 2: Figure S2 — (A) Cell fractionation reveals the presence of GFP-Sec7 in the cytosol and in membrane fractions. The cells were opened using Nuclepore filters and aliquots separated by SDS PAGE (10% acrylamide). Proteins were detected with mAb K3-184-2 [47]. L, whole cell lysate, P0 (400 × g); SN1, P1 (10,000 × g); SN2, P2 (100.00 × g). SN, supernatant; P, pellet. The signal obtained for whole cell lysate, 10,000 × g pellet and supernatant and 100,000 × g supernatant represents the material from 2×10^5^ cells/ml, the signal in the 100,000 × g pellet corresponds to 2×10^7^ cells/ml. (B) Coronin and proteasomal subunit interact with GFP-Sec7. GFP-Sec7 was immunoprecipiated using GFP antibodies and the pull down probed for the presence of coronin using mAb 176-3-6 and mAb 159-83-10 to detect the 28 kDa proteasomal subunit psmA4 (DDB0214953). psmA4 was observed in one pull down experiment only and was therefore not included in Table 1. Here it proved to be a binding partner. For control, sec7- expressing GFP-LimD was used. (C) Coimmunoprecipitation of GFP-Sec7 with coronin. Protein A sepharose beads carrying mAb 176-3-6 were used to precipitate coronin from cell lysates of sec7- cells expressing GFP-Sec7 or GFP-LimD and the immunoprecipitates probed for the presence of GFP-Sec7 using GFP-specific antibodies. [file 1478-811X-11-54-S2.pdf]

## Slide 1
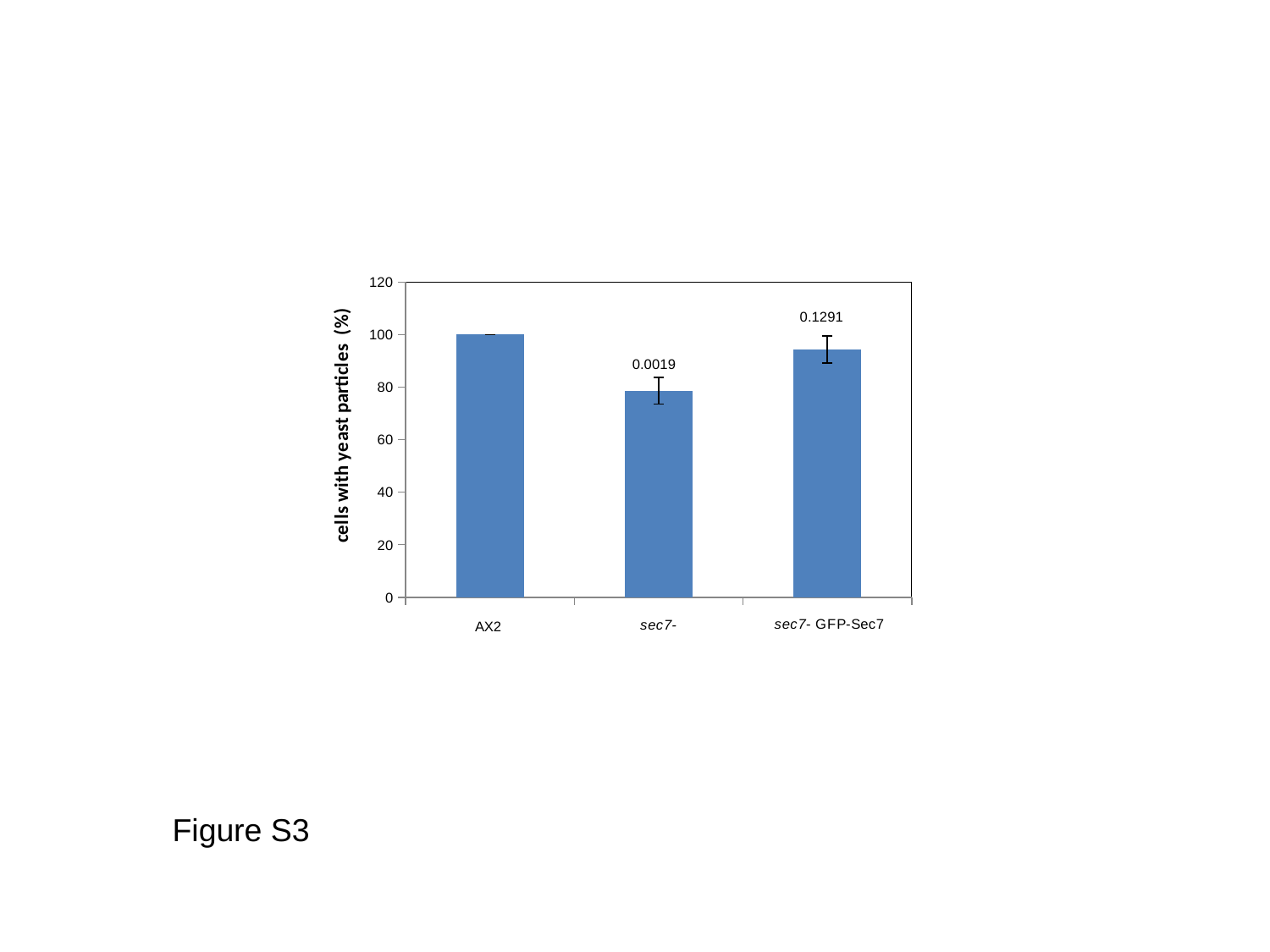

### Chart: 0.1291
| Category | |
|---|---|
| | 100.0 |Figure S3

Supplement: Additional file 4: Figure S3 — sec7- cells have a phagocytosis defect. AX2, sec7- and sec7- expressing GFP-Sec7 cells were incubated with yeast and fixed after 45 minutes. The number of cells containing yeast particles was determined. The results shown are from three independent experiments. P values are given. The difference between sec7- and AX2 is significant. [file 1478-811X-11-54-S4.pptx]
