# Supplementary material for: The cytohesin paralog Sec7 of Dictyostelium discoideum is required for phagocytosis and cell motility
Source: Cell Commun Signal. 2013 Aug 1;11:54. doi: 10.1186/1478-811X-11-54 (PMC3737031; doi:10.1186/1478-811X-11-54)
Supplement: Additional file 3: Table S1 — GO classification of the Sec7 interaction partners and Table S2 Mutant phenotypes of potential Sec7 interaction partners. The GO analysis was carried out at http://go.princeton.edu/cgi-bin/GOTermMapper. [file 1478-811X-11-54-S3.docx]

Table S1. **GO classification of the Sec7 interaction partners.** The analysis was carried out at http://go.princeton.edu/cgi-bin/GOTermMapper.

| **GO annotation** | **GFP-Sec7** | **Sec7 domain** | **PH domain** |
| --- | --- | --- | --- |
| transport  ( GO:0006810 ) | 34 of 37 genes, 91.89% | 18 of 24 genes, 75.00% | 18 of 21 genes, 85.71% |
| vesicle-mediated transport  ( GO:0016192 ) | 32 of 37 genes, 86.49% | 18 of 24 genes, 75.00% | 17 of 21 genes, 80.95% |
| biosynthetic process  ( GO:0009058 ) | 10 of 37 genes, 27.03% | 4 of 24 genes, 16.67% | 5 of 21 genes, 23.81% |
| small molecule metabolic process  ( GO:0044281 ) | 10 of 37 genes, 27.03% | 6 of 24 genes, 25.00% | 5 of 21 genes, 23.81% |

Table S2. **Mutant phenotypes of potential Sec7 interaction partners.**

| **Mutant** | **Defective processes** | | | | | |
| --- | --- | --- | --- | --- | --- | --- |
|  | phagocytosis | growth | cytokinesis | chemotactic motility | development | secretion |
| sec7 | + | + |  | + |  | ± |
| gapA |  |  | + |  |  |  |
| filamin | + |  |  | + |  |  |
| coronin | + | + | + | + | + |  |
| adaptin |  | + | + |  | + |  |
| arpC, arpB |  |  |  | + |  |  |
| smlA |  |  |  |  |  | + |
| zizB |  |  | + | + | + |  |
| myoI | + |  |  |  |  |  |
| cortexillin I |  |  | + |  |  |  |

+, indicates a defect
